# Supplementary material for: Diverging Arabidopsis populations quickly accumulate pollen-acting genetic incompatibilities
Source: Evol Lett. 2025 Jun 3;9(4):461–72. doi: 10.1093/evlett/qraf013 (PMC12448202; doi:10.1093/evlett/qraf013)
Supplement: qraf013_suppl_Supplementary_Figure_S1 [file qraf013_suppl_supplementary_figure_s1.pdf]

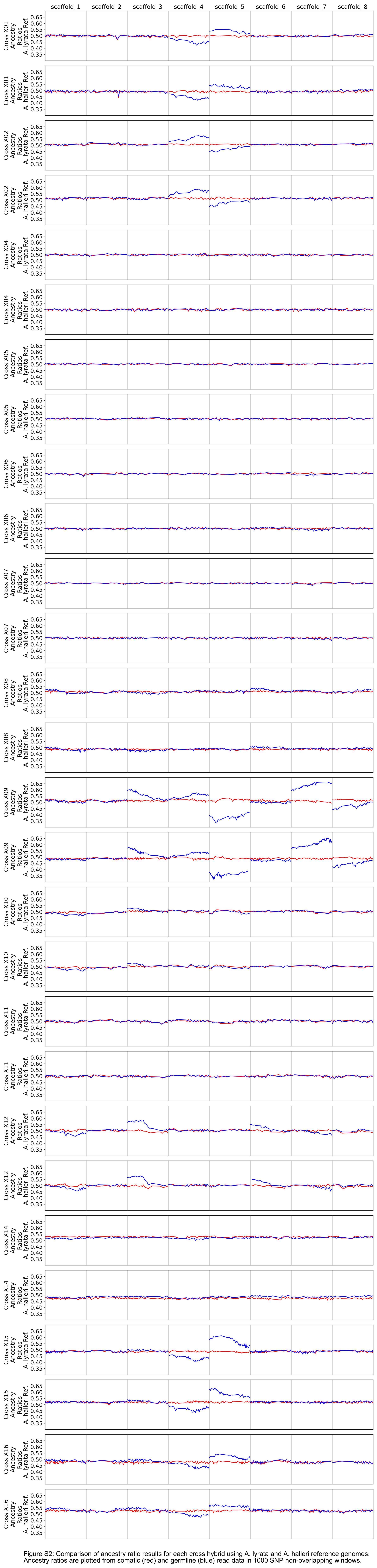

Figure S2: Comparison of ancestry ratio results for each cross hybrid using A. lyrata and A. halleri reference genomes. Ancestry ratios are plotted from somatic (red) and germline (blue) read data in 1000 SNP non-overlapping windows.
