# Supplementary material for: Diverging Arabidopsis populations quickly accumulate pollen-acting genetic incompatibilities
Source: Evol Lett. 2025 Jun 3;9(4):461–72. doi: 10.1093/evlett/qraf013 (PMC12448202; doi:10.1093/evlett/qraf013)
Supplement: qraf013_suppl_Supplementary_Table_S2 [file qraf013_suppl_supplementary_table_s2.pdf]

Cross	Species	Cross Type	Region Type	Mother	Father	Parent Genetic Dist. (π)	Parent Dist. (km)	Chr1 Effect Size	Chr2 Effect Size	Ch3 Effect Size	Chr4 Effect Size	Chr5 Effect Size	Chr6 Effect Size	Chr7 Effect Size	Chr8 Effect Size
WR06	halleri	within species	Within	Mias05 (POL)	Zapa07 (POL)	0.0043	154.9	0	0	0	0	0	0	0	0
WR07	halleri	within species	Within	Klet02 (POL)	Kowa07 (POL)	0.0043	91.1	0	0	0	0	0	0	0	0
WR04	halleri	within species	Within	Noss10 (ITA)	Pais04 (ITA)	0.0049	35.8	0	0	0	0	0	0	0	0
WR05	halleri	within species	Within	Pais04 (ITA)	Noss10 (ITA)	0.0049	35.8	0	0	0	0	0	0	0	0
BR01	halleri	within species	Between	Pais09 (ITA)	Wall02 (DEU)	0.0052	494	0	0	0	-0.07	0.06	0	0	0
BR02	halleri	within species	Between	Wall10 (DEU)	Pais09 (ITA)	0.0052	494	0	0	0	0.07	-0.05	0	0	0
BR12	lyrata	within species	Between	70544 (NOR)	Alyr5a (DEU)	0.0052	1322.7	-0.05	0	0.1	0	0	0.04	-0.02	0
BR10	lyrata	within species	Between	70536 (NOR)	Alyr6a (DEU)	0.0054	1322.7	-0.02	0	0.02	0	-0.02	0	0	0
BR11	lyrata	within species	Between	70535 (NOR)	79b (DEU)	0.0055	1322.7	0	0	0	0	0	0	0	0
BS09	hybrid	between species	N/A	Al: LF10 (AUT)	Ah: Hall2.2 (ITA)	0.0080	451	0	0	0.1	0.06	-0.19	-0.02	0.19	-0.08
BS08	hybrid	between species	N/A	Al: 79b (DEU)	Ah: Hall2.2 (ITA)	0.0084	425.2	0	0	-0.02	0	0	0.02	0	0
BS15	hybrid	between species	N/A	Al: CP1-1 (CZE)	Ah: I14 (ITA)	0.0087	567.5	0	0	0.02	-0.08	0.13	0	0	0
BS16	hybrid	between species	N/A	Al: CP991 (CZE)	Ah: I16 (ITA)	0.0088	566.5	0.02	0.02	0.03	-0.05	0.06	0	0	0
BS14	hybrid	between species	N/A	Al: MN (USA)	Ah: Lan5 (DEU)	0.0090	6948.1	-0.02	0	0	-0.02	0	0	0	0
